# Using artificial intelligence to identify the top 50 independent predictors of subjective well-being in a multinational sample of 37,991 older European & Israeli adults

**DOI:** 10.1038/s41598-023-38337-w

**Published:** 2023-07-13

**Authors:** Germano Vera Cruz, Thomas Maurice, Philip J. Moore, Cynthia A. Rohrbeck

**Affiliations:** 1 UR 7273 CRP-CPO, Department of Psychology, University of Picardie Jules Verne, Bât E-1, Chemin du Thil, 80025 Amiens, France; 2 EA 2249 CRIEF, Department of Health Economics, University of Poitiers, Poitiers, France; 3 Department of Psychological and Brain Sciences, The George Washington University, Washington, DC USA

**Keywords:** Psychology, Health occupations

## Abstract

Subjective well-being (SWB) is widely recognized as an important health outcome, but its complexity, myriad predictors, and analytic requirements pose significant challenges to identifying the relative order and impact of SWB determinants. This study involved a representative sample of 37,991 older adults from 17 European countries and Israel. An aggregate index of SWB was developed and compared across countries, and machine-learning algorithms were used to rank-order the strongest 50 (of an initial 94) SWB predictors from 15 categories. General Additive Modeling (GAM) and low-degree polynomials (i.e., splines) were used to determine the independent effect sizes and significance levels for each of these top-50 SWB predictors. Of the 18 countries included in this study, Denmark had the highest mean SWB, while Greece had the lowest. The two top-ranked SWB predictors (loneliness, social activity satisfaction) were social factors, which also had the highest overall group ranking, followed by physical health, demographics, financial status and personality. Self-reported health was the strongest health-related predictor, neuroticism was the strongest personality predictor, and women reported higher SWB than men. SWB decreased with age, and increased with income up to 350,000 euros/year, after which it declined. Social factors were of primary importance for subjective well-being in this research, while childhood experiences and healthcare status exerted the smallest effects. The vast majority of the top 50 SWB predictors were statistically significant, with the notable exceptions of body mass index and most health behaviors, which may impact SWB indirectly through their effects on physical health. Future multivariate modeling is recommended to clarify the mechanisms for these and other observed relationships.

## Introduction

Subjective well-being (SWB) is increasingly recognized as an essential component of human health^1^, a valuable marker of social progress^2^, and a key piece of information for public policy^3^. An important outcome in its own right, greater SWB is also associated with greater productivity across occupations^4^, less illness, and longer lives^5^. These benefits are particularly important for older adults (e.g., 50 years and older), who represent an increasingly large percentage of the world’s population^6^. Accordingly, SWB has become an increasingly prominent topic of research, as indicated by an electronic literature search (PsycINFO, APA PsycArticles, MEDLINE, & Academic Search Complete) in April 2023 that identified 735 articles with “subjective well-being” in the title between 1980 and 2001, and over 7600 such articles since 2002. Further understanding, improvement and utility of SWB involves addressing a number of ongoing questions considered in the literature and elsewhere.

First, what is subjective well-being, and how can it be assessed? Subjective well-being is a broad, multidimensional construct that refers to how people evaluate and experience specific activities, general domains, and the overall state of their lives^7^.

*Evaluative* SWB refers to individuals’ perceived life quality, fulfillment or satisfaction, and its measurement includes single-item evaluations and aggregate assessments of multiple dimensions. An example of an overall, single-item assessment of evaluative well-being is “Overall, how satisfied are you with life as a whole these days?”, which has been used in many social surveys in Europe and North America^8^. The Cantril Self-Anchoring Striving Scale^9^—also used in many worldwide satisfaction surveys—has respondents indicate their position on a ladder from their “worst possible life” to “best possible life,” and is often used for current and projective life-satisfaction assessments^10^. Among the most prominent compound measures of life satisfaction is the Satisfaction with Life Scale (SWLS^11^), a well-validated Likert scale on which people indicate their agreement with five life statements (e.g., “So far, I have gotten the important things I want in life”). Apart from life satisfaction, a more recent index of evaluative SWB is the 19-item Quality of Life Scale (CASP-19)^12^, a quality-of-life (QoL) measure designed to assess perceptions of control, autonomy, self-realization and pleasure among older adults, and which is increasingly used in research on aging^13^.

*Experiential* SWB refers to the frequency and intensity of emotional experiences that make a person’s life more pleasant or unpleasant^14^. Positive affect has long been associated with better physical health^15,16^ and survival among older adults^17^. Perhaps the best-known example of positive experiential SWB is the World Happiness Report (WHR), which includes an annual ranking of happiness in over 140 countries^18,19^.

Similarly, negative emotion—particularly depression—has significant effects on health-related behaviors and quality of life^20,21^, as well as future physical functioning^22^. The distinction between positive and negative affect is crucial, both because they are independent predictors of life satisfaction and other outcomes^7^, and because the value of experiential SWB research lies not only in enhancing positive experiences, but also in reducing the suffering associated with negative ones.

The significant number of SWB-related factors suggests the value of aggregate SWB measures for improving our insight into subjective well-being. However, the prevalence of these factors also poses an additional challenge to better understanding SWB and its potential causes and effects. For while combining related elements can provide more information about the construct, it can also make it more difficult to clarify what SWB actually *is*, or to distinguish it from its antecedents and consequences. As concluded by the National Research Council^7^, “… the components of SWB display distinct characteristics, often correlate with different sets of variables, and capture unique aspects of the construct that for various purposes are worth monitoring”. [However,] The terms used to describe SWB have often been ambiguously applied, which has muddled discussion and possibly slowed progress in the field.” (section 1.1).

On the other hand, this ambiguity is understandable, given the many fields, approaches and purposes for which SWB is assessed. As Eid and Larsen^23^ point out, how SWB is conceptualized and operationalized is often dictated by the nature of the topic, setting, or research question of interest. For example, economists may include utility derived from material resources^24^, while public health researchers may consider aspects of physical health such as pain or illness symptomology^25^. Nonetheless, the increased complexity and ambiguity associated with aggregate SWB measures also argue for specifying the content, rationale, and precedent (if any) of these indices, and how they compare with other potential measures of SWB.

Second, how is subjective well-being distributed internationally, and what can we learn from this? Measures of SWB have long been assessed across countries^26^, and have included life satisfaction^27^, quality of life^28^, happiness & unhappiness^29^, anxiety^30^ and depression^31^, among others. The results of these and other studies of SBM components suggest that, as a whole, SWB varies widely across countries, and that, as predicted over 35 years ago^26^, self-reported measures of SWB “can be expected to contribute substantially to our understanding of the causes and conditions of individual well-being.” (p.2). Assessing SWB across countries can also help to identify vulnerable populations, and provide further insights into how to improve SWB at the national level, and testing the statistical significance between SWB predictors can discern more empirically-meaningful differences between these countries, especially in studies with representative samples.

Third, what factors predict SWB at the individual level, and for older adults in particular? Individuals’ SWB is influenced by many factors, including demographics, socioeconomic status, physical environment, working conditions, social factors, mental & physical functioning, personality, and dozens of others^32–36^. For example, age, gender and marital status have been significantly associated with life satisfaction^35^, as have education, income, and social capital^37,38^. SWB may also be a function of personality traits like optimism, which is consistently and positively associated with satisfaction^39^, and neuroticism, which is a strong, negative predictor of SWB^40^. However, given the sheer number and predictive overlap of these factors, it is difficult to determine their unique, relative impact on subjective well-being. As a result, SWB predictors have typically been assessed in relatively small combinations, at different times, and with different indices.

Just as the psychosocial profiles and patterns of children & adolescents can differ markedly from those of adults^41^, so too can these outcomes and relationships differ between older adults and younger individuals^42,43^. In addition, the relative dearth of research on older adults^44^ and their increasing numbers throughout the world also indicate the importance of research on this population.

Finally, how can dozens of prospective SWB predictors be independently assessed? Depending on the effect sizes, significance levels and other parameters, ensuring the power necessary to assess the unique impact of 50 or more factors may require thousands of participants. For example, using a classic rule of thumb [*n* = *k*(*m* + *1*)] based on the Central Limit Theorem (i.e., that a distribution of sample means is essentially normal with 30 or more observations), a minimum sample size (*n*) for the initial 94 predictors in this research (*k),* 30 observations per variable (*m*), and a Bonferroni correction for 20 tests would be 3680^45^. Large sample sizes are particularly important in applied, observational contexts (e.g., surveys, field settings), which typically involve more measurement error, and from which such data are more likely to be obtained. It is also important to use methodologies that can (1) identify patterns within extremely large datasets, (2) provide unbiased factor selection and prioritization, and (3) increase predictive accuracy by minimizing error variance.

To address these questions, the current research involved almost 38,000 older adults randomly selected within 18 countries, an aggregate measure of subjective well-being, and 94 prospective SWB predictors. Factor analysis was conducted to create the aggregate SWB measure, and artificial-intelligence modeling (machine learning) was used to rank-order the top 50 potential predictors of SWB—from 15 categories—in terms of error reduction. General Additive Modeling (GAM^46,47^) and Analysis of Variance (ANOVA) were then used to determine the effect sizes and statistical significance of these 50 predictors. Thus, this research is designed to contribute to the field by utilizing machine learning to empirically assess and compare the independent effects of dozens of factors on a combined (evaluative and experiential) measure of SWB among thousands of older adults from several countries.

## Methods

### The study data

The data for this study come from the Survey on Health Aging and Retirement in Europe (SHARE) survey, a large-scale, ongoing program developed, administered and maintained by a multidisciplinary team of researchers, clinicians and statisticians in 26 European countries and Israel^48^. The SHARE survey has been conducted since 2004, and includes longitudinal interviews with representative samples of European and Israeli adults aged 50 and older^49,50^. Participants are typically interviewed every two years until their death, at which point an end-of-life survey is offered to their relatives to obtain information about the end of participants’ lives. With eight waves of data from over 140,000 participants, the SHARE survey is one of the most comprehensive studies of aging in the world.

Because Wave 7 was a “special edition” (due to its emphasis on participants’ childhoods), the data in the current study includes Waves 6 & 7 (2014 & 2016) to provide past and present information about participants. The data in this study were collected from 17 of the 26 European Union (EU) countries (Austria, Belgium, Croatia, Czech Republic, Denmark, Estonia, France, Germany, Greece, Italy, Luxembourg, Poland, Portugal, Slovenia, Spain, Sweden & Switzerland) and Israel, and includes a total of 37,991 participants. These participants—21,412 (56.4%) of whom were female—ranged in ages from 50 to 102 years, with a mean age of 66.1, a median age of 65, and a standard deviation of 9.7 years.

### Subjective well-being

The SHARE data in the current research was developed, collected and analyzed by an interdisciplinary team of research scientists and clinicians. Accordingly, the current measure of subjective well-being was created by combining participant responses to three indices of SWB. The first component is a single-item assessment of life satisfaction, which asked participants to respond to the question, “All things considered, how satisfied are you with your life as a whole,” on a scale from 0 (completely dissatisfied) to 10 (completely satisfied). This overall, single-item index has been used extensively to assess life satisfaction^51^, and its construct validity has been affirmed by its significant association with responses to the SWLS^52^. This measure was also included because of its positive contribution to subjective well-being, and because it is among the most ubiquitous SWB components across fields^23^.

The second component is the 12-item Quality of Life Scale (CASP-12), a shortened version of the CASP-19 quality-of-life (QoL) measure^53^, in which participants indicated how often—from 0 (never) to 3 (often)—they have had each of 12 experiences (e.g., I look forward to each day”). Responses to negative items (e.g., “I feel left out of things”) were reverse-coded, after which all responses were combined for a possible overall score of 0 to 36 for each participant, with higher scores indicating higher QoL. In addition to a solid factor structure and internal reliability (Cronbach’s alpha = 0.86), this scale was included in this SWB measure because of its strong association with subjective well-being and its widespread use in research among older adults^7,53^.

The third SWB component in this research is depression, as assessed by the Euro-D depression scale, a shortened version of the original 14-item Euro-D^54^. Participants responded to this scale by indicating whether or not (1 or 0, respectively) they had experienced each of 12 depressive symptoms during the previous month, resulting in potential overall scores of 0 to 12, with higher scores indicating more depression. These overall Euro-D scores were reverse-coded before being combined with the other two SWB components. It is increasingly clear to that positive and negative aspects of SWB do not simply operate as a single continuum^55^, indicating the importance of including negative SWB components, which is still relatively rare^7^. Depression was included in this SWB measure because of it significant negative impact on SWB^56^, and because of the Euro-D’s strong factor structure and internal reliability (Chronbachs > 0.80)^57^.

A principal component factor analysis was conducted on the three SWB component scores using SPSS 28.00. This unrotated analysis yielded the following factorial structures: Component 1 (eigenvalue) = 2.03; Component 2 (eigenvalue) = 0.57; and Component 3 (eigenvalue) = 0.39. Component 1—which was used for the current compound SWB measure—explained 67% of the total variance, and correlated significantly with life satisfaction, QoL, and depression (0.87, 0.81, and -0.79, respectively). In addition, assumptions of adequacy (KMO = 0.672) and sphericity (Bartlett’s (ddl = 3) = 27,294.815, p ≤ 0.001) were also met. The items for this measure were then centered, standardized, and averaged to create a composite index of subjective well-being (mean = 0, median = 0.17, SD = 0.97, min = −4.98, max = 1.67).

This three-part compound SWB measure has been used in a number of previous SHARE studies^33,58,59^, and it represents an attempt to maximize content validity (by assessing multiple domains within both evaluative and experiential well-being), while minimizing participant burden by using the shortest available versions of the relevant scales. Of course, there are different ways to conceptualize SWB and its components. Thus, to put the current SHARE SWB measure in a broader context, Table 1 presents examples of how different fields approach and operationalize subjective well-being and related factors.

**Table 1:** Factors related to subjective well-being across disciplines.

Discipline	SWB-related approaches	SWB-related constructs	SWB-related measures	Citations^a^
Economics	– Frequent emphasis on utility from material resources – Can also include income (esp. relative income)	– Utility – Income – Happiness – Unhappiness – Life satisfaction	– Satisfaction with Life Scale (SWLS) – World Values Survey (WVS) – U-Index – General Social Survey (GSS)	Benjamin et al., 2017 Diener et al., 1985 Easterlin, 1995 Frey & Stutzer, 2002 Kahneman & Krueger, 2006
Sociology	– Unit of analysis is often groups (e.g., families) – Often focused on links and overlap between SES, demographics, and SWB – Quality of life is often the primary outcome of interest (with SWB as part to it)	– Meaning – Quality of life – Life satisfaction – Group disparities	– Cantril Self-Anchoring Striving Scale (CSASS) – Satisfaction with Life Scale (SWLS) – General Social Survey (GSS) – World Happiness Report (WHR)	Diener et al., 1985 Diener et al., 2009 George, 2010 Kelly & Evans, 2017 Veenhoven, 2008
Psychology	– Unit of analysis is typically the individual – Strong emphasis on testing multi-item SWB-related scales – Scales used also depend on area (e.g., stress, personality, positive psychology) – Methodology includes ecological momentary assessment	– Meaning – Quality of life – Life satisfaction – Stress & coping – Happiness – Unhappiness – Anxiety, and depression	– Positive and Negative Affect Schedule (PANAS) – Perceived Stress Scale (PSS) – Satisfaction with Life Scale (SWLS) – State-Trait Anxiety Inventory (STAI) – Beck Depression Inventory (BDI)	Diener et al., 1985 Kashdan, 2004 Gillham et al., 2011 Goodman et al., 2018 Lyubomirsky & Dickerhoof, 2005 Stone & Mackie, 2013
Public health	– Need/goal engagement & fulfillment as theoretically central, linking orientations and SWB outcomes – Most SWB measures contain evaluative or experiential measures, but not both – Emphasis on link between SWB and physical health	– Quality of life – Life satisfaction – Mental and physical functioning – Positive and negative emotion	– Short-Form Health Survey (SF-36) – Satisfaction with Life Scale (SWLS) – Health-Related Quality of Life Scale (HRQOL) – Physical Activity Scale for the Elderly (PASE)	CDC, 2023 Das et al., 2020 Diener et al., 1985 Diener et al., 2018 Ryff & Keyes, 1995
Public policy	– SWB often based on public’s self-reported life satisfaction, assessed in response to policy – Unit of analysis is typically large, including countries	– Life satisfaction – Institutional trust – Social capital – Development indices (for individuals and populations)	– General Trust Scale (GTS) – Personal Social Capital Scale (PSCS) – Satisfaction with Life Scale (SWLS) – Human Development Index (HDI)	Diener et al., 1985 Dolan et al., 2008 Dolan & Metcalf, 2012 Odermatt & Stutzer, 2018 Vik & Carlquist, 2018
SHARE	– An inter-disciplinary approach to SWB-related factors and measurement – Combines single & multi-item, positive & negative, and evaluative & experiential SWB measures	– Life satisfaction – Quality of life – Depression	– Single-item life satisfaction measure – Control, Autonomy, Self-realization, and Pleasure (CASP-12) – EURO-D Depression Scale	Jovanović, 2020 Pérez-Rojo et al., 2018 Prince et al., 1999

^a^The reference list is in Appendix C.

### SWB predictors

#### Predictor categories

The SWB predictors in this study came from 15 categories representing different domains of human experience. These categories (with the number of predictors in parentheses) include: (1) Demographics (7), (2) Family Status (5), (3) Societal Factors (5), (4) Childhood Experiences (9), (5) Living Environment (7), (6) Work Environment (3), (7) Financial Status (9), (8) Social Factors (6), (9) Physical Health (7), (10) Mental Health (2), (11) Cognitive Function (3), (12) Healthcare (11), (13) Health Behaviors (11), (14) Personality (5), and (15) Future Expectations (4). While the majority of these categories come from previous studies using SHARE data^48–50^, categories 2, 3, 5, and 15 were based on other research demonstrating links between these categories and SWB^40,59–64^. Not all of these categories were represented in all of these studies, nor did their individual predictors overlap completely with those in the current research. However, all of the previous predictors within the categories that correspond to those in the current study were significantly associated with SWB. This approach enables both direct comparisons with previous SHARE findings, while also providing results to address additional current and future SWB issues.

#### Predictor variables

Given the exploratory nature of this study, all variables in the SHARE data that were relevant to the predictor categories were included in the initial set of predictors. In addition to standard category exemplars, this initial set of 94 variables (listed in Appendix A) also included less common predictors extracted from the SHARE database. For example, beyond age and gender, demographic predictors included current participants’ nationality and country of birth, while financial status included not only income, but also participants’ ability to make ends meet. Given their conceptual overlap, many SWB predictors were relevant to more than one category. Thus, category designations were made in accordance with the primary themes of the research. For example, childhood loneliness was included in childhood experiences, periods of hunger were considered a marker of financial status, and current loneliness was categorized as social relationship factors.

While most predictors were kept in their original form, some required transformations and/or aggregation by the SHARE research team. Specifically, due to the significant number of missing values for participant income, data imputation was conducted for income-related variables. This imputed income variable was then adjusted for the relative prosperity of each country, and the exchange rate between their national currency and the euro in 2015, allowing for more valid comparisons. For the same reason, education was assessed using the International Classification of Education (ISCED-97 classification), which related educational attainment in each country to an international standard. Finally, to clarify the descriptive results and directionality of effects, the original SHARE scales worded in the opposite direction indicated by the measure were reverse-coded in the present study, and dichotomous predictors were recoded as 0/1, with 1 reflecting more of the predictor (see Appendix A and B). As confirmed in subsequent analyses, this reverse-coding—while changing the valence—had no effect on the strength of the associations between any of the study variables.

### Data analysis

#### Rank-ordered predictors

To rank-order the 50 strongest SWB predictors, we used machine learning, which is an application of artificial intelligence whose algorithms build models through iterative analyses of sample data to generate statistical predictions that they are not specifically programmed to test. First, we used the “VSURF” program within the statistical package R^65^, whose iterative regression modeling was used to identify the independent, multivariate contribution of each variable to the overall explained variance (R^2^) for the model predicting SWB. After this selection process was completed, a machine-learning regression model was built using the Random Forest algorithm^66^.

The Random Forest algorithm (which was also used to perform the missing-data imputations) uses a random subset of predictors to test the strength of each predictor in a model through a process called *recursive partitioning*. This process involves first developing a decision tree from the strongest available predictors, and then testing the tree’s overall predictive power on a subset of data not used to construct the tree itself (also called “out of bag” sampling). The Random Forest algorithm does this repeatedly, bootstrapping up to thousands of decision trees, and then averaging their results.

In the current study, each Random Forest model was constructed from 500 regression trees, with the number of predictors available for splitting at each tree node equal to one-third the number of predictors. Among other outputs, this process yielded the percent increase in mean squared error (MSE) for each factor in the model predicting SWB, which is the percentage increase in the MSE caused by removing that factor from the model. As such, this measure reflects the extent to which each factor reduces the difference between the predicted and actual SWB values, with higher values indicating stronger, more accurate predictors of SWB, which were then used to rank-order the top 50 SWB predictors.

In machine learning, the original dataset is split into at least two sets: one to train the model (usually 70–80% of the sample), and the other to estimate its predictive performance (usually 20%-30% of the sample). In the current study, the training set constituted 70% of the sample, while the testing set comprised 30%. The final model predicted a majority of the variance in SWB (R^2^ = 58.71%), with very low residual error (RMSE = 0.41). However, because these MSE analyses do not include main effects or inferential results, additional analyses were conducted to determine the effects sizes and significance levels for each of the top 50 SWB predictors.

#### Effect sizes & significance

To determine the effect sizes and significance of the SWB predictors in this research, we applied Generalized Additive Modeling (GAM) to the continuous and ordinal data^46,47^, while nominal predictors were subjected to Analysis of Variance (ANOVA). A GAM algorithm was chosen because GAMs are better able to fit nonlinear data. Moreover, compared with Generalized Linear Models (GLMs) such as linear regression, GAMs do not assume that the predictive relationship is a simple weighted sum, but rather that it can be modeled by a sum of arbitrary functions of each feature^47^. In GAM, the beta coefficient from linear regression is replaced with a flexible function that enables the assessment of non-linear relationships. This flexible function—called a *spline*—is a piecewise polynomial that fits multiple, low-degree polynomials to small subsets of values. A primary advantage of splines relative to high-degree polynomials is that they reduce statistical error by reducing the variability between interpolation points^67^. Relative to GLMs, GAMs are also better able uncover patterns in the data.

The general equation for GAM can be expressed as follows:

$${\text{F}}\left( {\text{x}} \right) = {\text{yi}} = \alpha + {\text{f}}_{{1}} ({\text{xi}}_{{1}} ) + {\text{f}}_{{2}} ({\text{xi}}_{{2}} ) + \ldots .{\text{f}}_{{\text{p}}} ({\text{xi}}_{{\text{p}}} ) +\upvarepsilon {\text{i}}$$

where f_1_, f_2_, f_3_, …. f_p_ are different non-linear functions on variables X_p_. In essence, GAM is a broader, more comprehensive modeling approach that can incorporate non-linear functions— using splines, step functions, etc.—while retaining the ability to test simpler, additive models as well^47^. In this research, multiple GAM models were generated using different fitting parameters, including *families* (i.e., groups of data-modifying functions), *knots* (i.e., the number of spline nodes), *fitting methods* (i.e., model component estimation algorithms), and *optimizers* (curve-smoothing selection algorithms). The final model—which resulted in the best-fit restricted maximum likelihood (REML)—explained over 55% of the variance in participants’ subjective well-being.

### Ethics approval and consent to participate

No applicable. The dataset used in this study come from the raw data collected in the SHARE survey framework. The institutions responsible for the SHARE survey are the ones who dealt with the ethical issues.

## Results

### SWB across countries

Subjective well-being varied widely across the countries included in this research. As shown in Table 2, national sample sizes range from 340 (Poland) to 2982 (Belgium), with the highest SWB score coming from Denmark (0.575), and the lowest from Greece (-0.645), and a significant overall eta-squared of ɳ^2^_p_ = 0.301, p < 0.0001. In addition, post-hoc Bonferroni specific-comparison tests indicated eight tiers of countries—a through h—that differed significantly in terms of SWB, including, in order, Denmark/Switzerland, Sweden/Austria, Luxembourg/Germany, Belgium/Slovenia/Spain, Spain/France/Czech Republic/Israel, Italy/Croatia/Poland, Poland/Estonia/Portugal, and Greece. When examined regionally (relative to Switzerland), Northern European countries’ SWB (M = 0.282) was significantly higher than that in Southern Europe (M = −0.320) (t(8) = 4.34, p < 0.01), while Eastern European countries’ SWB (M = −0.062) did not differ significantly from either of the other two regions. In addition, the rank-order of countries’ SWB in this research is also very similar to their rank order in the World Happiness Report in 2022 and 2023 (r(18) = 0.89, p < 0.0001)^18,19^.

**Table 2:** Descriptive and inferential analysis for SWB across countries.

Country*	N (%)	M	SD	Overall results
Denmark^a^	1783 (4.7)	0.575	0.789	All countries *F*(17, 37,973) = 223.120, *p* < 0.0001, ɳ^2^_p_ = 0.301 North (Denmark, Sweden, Luxembourg, Germany, Belgium) M = .282^x^ South (Spain, Italy, Croatia, Portugal, Greece) M = −0.320^y^ East (Austria, Slovenia, Czech Republic, Poland, Estonia) M = −0.062^x,y^
Switzerland^a^	1531 (4.0)	0.499	0.748	
Sweden^b^	1958 (5.2)	0.386	0.770	
Austria^b^	2194 (5.8)	0.376	0.845	
Luxembourg^c^	1072 (2.8)	0.212	0.900	
Germany^c^	2756 (7.3)	0.208	0.872	
Belgium^d^	2982 (7.8)	0.030	0.913	
Slovenia^d^	3383 (8.9)	0.030	0.937	
Spain^d,e^	2952 (7.8)	−0.040	0.958	
France^e^	1875 (4.9)	−0.070	0.915	
Czech Republic^e^	2933 (7.7)	−0.073	0.858	
Israel^e^	1575 (4.1)	−0.100	0.857	
Italy^f^	2594 (6.8)	−0.227	1.020	
Croatia^f^	1967 (5.2)	−0.242	1.131	
Poland^f,g^	340 (0.9)	−0.274	1.025	
Estonia^g^	4579 (12.1)	−0.371	0.980	
Portugal^g^	482 (1.3)	−0.448	1.011	
Greece^h^	1035 (2.7)	−0.645	1.063	

*N* sample size, *M* standardized SWB index mean, *SD* standard deviation.

*Countries and regions without a common superscript have significantly different levels of SWB (p < 0.05).

### Rank-ordered SWB predictors

Forty-eight of the 50 top SWB predictors included 37,991 observations (see Table 3). Three of the top 10 SWB predictors—including the top two—were social factors, three were aspects of physical health, two reflected financial status, and one each came from demographic and personality categories. Loneliness was the top overall SWB predictor (M = 0.88 on a 0–6 scale), followed by social activity satisfaction (#2, M = 8.00 out of 10) and social network satisfaction (#7, 8.94 out of 10), respectively. Participants reported an average general health (#3) of 2.84 (out of 5), an average of 1.75 chronic diseases (#10), and a 46% rate of limited activity due to health (#9). Financially, participants also reported being able to make ends meet most of the time (#4, M = 2.89 out of 4) with an average income (#8) of 98,570 euros and a median of 26,505 euros.

**Table 3:** Descriptive results for the top 50 SWB predictors among older-adult Europeans and Israelis.

SWB predictors	Rank	Cat	*N*	Scale	*M* (%)	SD (s)
Loneliness	1	8	37,991	0–6	0.88	1.38
Social activity satisfaction	2	8	37,991	0–10	8.00	1.91
Self-rated general health	3	9	37,991	0–5	2.84	1.07
Making ends meet	4	7	37,991	1–4	2.89	0.85
Country	5	1	37,991	n/a	n/a	n/a
Neuroticism	6	14	37,991	1–5	2.62	0.98
Social network satisfaction	7	8	37,991	0–10	8.96	1.25
Income	8	7	37,991	0-2527K	99K	192K
Limited activity due to health	9	9	37,991	1–3	46% > 1	0.74
Number of chronic diseases	10	9	37,991	0–13	1.75	1.60
Alive in 10 years	11	15	37,991	0–100	68.25	28.51
Social contact frequency	12	8	37,991	0–7	4.81	2.17
Extraversion	13	14	37,991	1–5	3.51	0.92
No healthcare due to cost	14	7	37,991	Y/N	13.8%Y	n/a
Age	15	1	37,991	50–102	66.14	9.72
Social network distance	16	8	19,395	0–8	3.32	1.51
Social network size	17	8	19,395	0–7	2.69	1.48
Employment status	18	6	37,991	1–5	27.9%E	n/a
Vigorous physical activity	19	13	37,991	1–4	2.45	1.34
No heat due to cost	20	7	37,991	Y/N	6.9%Y	n/a
Have a chronic illness	21	9	37,991	Y/N	52.9%Y	n/a
Sex	22	1	37,991	F/M	56.4%F	n/a
Rooms at age 10	23	4	37,991	0–49	3.42	1.83
Conscientiousness	24	14	37,991	1–5	4.11	0.78
Moderate physical activity	25	13	37,991	1–4	3.44	1.00
Others helpful	26	5	37,991	1–4	3.17	0.61
Number of drinks in last 7 days	27	13	37,991	0–98	3.75	6.92
Happiness periods	28	10	37,991	Y/N	44.4%Y	n/a
Education	29	1	37,991	0–6	2.93	1.48
Books at age 10	30	4	37,991	1–5	2.27	1.23
Comprehensive insurance	31	12	37,991	Y/N	40.2%Y	n/a
Feel local connection	32	5	37,991	1–4	3.65	0.60
Agreeableness	33	14	37,991	1–5	3.70	0.79
Openness to experience	34	14	37,991	1–5	3.35	0.93
Long-term health insurance	35	12	37,991	Y/N	68.0%Y	n/a
Lonely in childhood	36	4	37,991	1–4	1.67	0.95
Informal care given	37	12	37,991	0–3	2.63	0.73
Frequency of prayer	38	3	37,991	1–6	2.60	1.79
Math skills at age 10	39	4	37,991	1–9	3.36	1.08
Looked after grandchildren	40	2	37,991	Y/N	21.5%Y	n/a
Body mass index	41	9	3799	13–75	27.11	4.62
Local help available	42	5	37,991	1–4	1.09	0.36
Physical abuse as child (father)	43	4	37,991	1–4	1.52	0.80
Physical abuse as child (nonparent)	44	4	37,991	1–4	1.26	0.63
Informal care received	45	12	37,991	0–3	2.74	0.60
Hunger periods	46	7	37,991	Y/N	5.3%Y	n/a
Private health insurance	47	12	37,991	Y/N	7.4%Y	n/a
Local crime a problem	48	5	37,991	1–4	1.87	0.70
Dairy consumption	49	13	37,991	1–5	4.39	1.05
Physical abuse as child (mother)	50	4	37,991	1–4	1.59	0.87

*N*, sample size; *M*, composite SWB index mean; SD, standard deviation; E, employed; n/a, not applicable; K, thousand; Y, Yes; N, Non; F, Females; M, Males.

Additional social SWB predictors in the top 50 included social contact frequency (#12), which averaged about three times a month (M = 4.81), social network distance (#16), which averaged a little over 1 km (M = 3.32), and social network size (#17), which averaged 2.69. Among other physical health factors, over half (52.9%) of participants reported having a chronic illness (#21), while their average body mass index (#41) was slightly overweight (M = 27.11). Financial status predictors also included the 13.8% of respondents who reported no healthcare due to cost (#14) and the 5.3% who reported periods of hunger (#46). All Big Five personality traits were among the top 50 SWB predictors, the strongest of which was neuroticism (#6, M = 2.62), followed by extraversion (#13, M = 3.51), conscientiousness (#24, M = 4.11), agreeableness (#33, M = 3.70), and openness to experience (#34, M = 3.35).

The bottom 10 SWB predictors included three childhood experiences, all of which reflected physical abuse by participants’ fathers (#43, M = 1.52 from 1 to 4), nonparents (#44, M = 1.26), or mothers (#50, M = 1.59). The two factors reflected respondents’ living environment included local help was available (#42, M = 1.09 from 1 to 4) and local crime was a problem (#48, M = 1.87). Within healthcare, most participants reported some informal care received (#45, M = 2.74 from 1 to 3), although relatively few of them (7.4%) reported having private health insurance (#47).

### Predicting SWB

Among the 50 strongest predictors of SWB, the percent increase in MSE ranged from a high of 174.19 (loneliness) to a low of 1.95 (physical abuse from mother in childhood), with a median value of 13.34 (for moderate physical activity). Only loneliness and social-activity satisfaction had MSE scores of over 100, while all remaining predictors had scores of less than 70 (see Table 4). In fact, the average contribution of these top two predictors to SWB was more than double that of any other predictor. Forty-two (84%) of the top 50 predictors had scores of less than 30, of which 6 (12%) were between 20 and 30, 17 (34%) were between 10 and 20, and 19 (38%) were below 10. All ten of the weakest predictors had scores of less than 6, and the weakest two (dairy consumption and physical harm from mother in childhood) had MSE scores of less than 3.

**Table 4:** Contribution, effect size and significance of the top 50 SWB predictors among older-adult Europeans and Israelis.

SWB predictors	Rank	Cat	%InMSE	+ /−	Effect (F)	P-value
Loneliness	1	8	174.19	−	2732.33	< 0.001
Social activity satisfaction	2	8	115.86	+	1334.08	< 0.001
Self-rated general health	3	9	69.42	+	898.99	< 0.001
Making ends meet	4	7	62.22	+	897.61	< 0.001
Country	5	1	59.06	n/a	223.12	< 0.001
Neuroticism	6	14	54.75	−	499.11	< 0.001
Social network satisfaction	7	8	47.01	+	166.47	< 0.001
Income	8	7	32.37	+ /−	13.37	< 0.001
Limited activity due to health	9	9	29.99	−	148.20	< 0.001
Number of chronic diseases	10	9	28.89	−	114.38	< 0.001
Alive in 10 years	11	15	27.11	+	169.11	< 0.001
Social contact frequency	12	8	22.82	+	3.86	< 0.05
Extraversion	13	14	20.65	+	36.53	< 0.001
No healthcare due to cost	14	7	20.26	−	1072.51	< 0.001
Age	15	1	19.65	−	25.39	< 0.001
Social network distance	16	8	19.65	−	5.60	< 0.05
Social network size	17	8	18.82	+	13.56	< 0.001
Employment status	18	7	16.97	+	15.29	< 0.001
Vigorous physical activity	19	13	16.05	+	6.14	< 0.01
No heat due to cost	20	7	15.89	−	1010.97	< 0.001
Have a chronic illness	21	9	15.64	−	2615.99	< 0.001
Sex	22	1	15.33	F > M	336.46	< 0.001
Rooms at age 10	23	4	14.41	+	7.40	< 0.01
Conscientiousness	24	14	14.29	+	111.44	< 0.001
Moderate physical activity	25	13	13.34	+	91.03	< 0.001
Others helpful	26	5	13.21	+	42.76	< 0.001
Number of drinks in last 7 days	27	13	12.27	+	0.16	0.69
Happiness periods	28	10	11.79	+	338.75	< 0.001
Education	29	1	11.41	+	18.55	< 0.001
Books at age 10	30	4	10.71	+	5.55	< 0.05
Comprehensive insurance	31	12	10.18	+	249.19	< 0.001
Feel local connection	32	5	9.87	+	54.13	< 0.001
Agreeableness	33	14	9.33	+	1.95	0.096
Openness to experience	34	14	8.81	+	4.19	< 0.05
Long-term health insurance	35	12	8.81	+	136.20	< 0.001
Lonely in childhood	36	4	8.64	−	4.81	< 0.01
Informal care given	37	12	8.45	+	35.81	< 0.001
Frequency of prayer	38	3	6.63	−	3.22	0.07
Math skills at age 10	39	4	6.41	+	3.29	< 0.05
Looked after grandchildren	40	2	6.33	+	116.36	< 0.001
Body mass index	41	9	5.99	−	3.15	0.059
Local help available	42	5	5.71	+	32.90	< 0.001
Physical abuse as child (father)	43	4	5.47	−	2.79	0.120
Physical abuse as child (nonparent)	44	4	5.04	−	2.94	< 0.05
Informal care received	45	12	3.73	+	15.03	< 0.001
Hunger periods	46	7	3.69	−	221.19	< 0.001
Private health insurance	47	12	3.23	+	0.235	0.628
Local crime a problem	48	5	3.05	−	7.15	< 0.01
Dairy consumption	49	13	2.73	+	0.27	0.605
Physical abuse as child (mother)	50	4	1.95	−	4.21	< 0.05

*N* sample size, *M* composite SWB index mean, *SD* standard deviation, *n/a* not applicable.

The majority (32) of the top 50 SWB predictors were significant at a p < 0.001 level, three predictors were significant at a p < 0.01 level, and 8 were significant at a p < 0.05 level. Two factors had p-values between 0.05 and 0.10, and five p-values did not approach statistical significance. Of the 43 significant predictors (i.e., p < 0.05), most (26) were positively associated with SWB (e.g., social activity satisfaction, self-rated health, extraversion), 14 were negatively related to SWB (e.g., loneliness, unable to make ends meet, neuroticism), two (country and sex) were not considered in terms of directionality, and one factor (income) had both positive and negative effects on SWB. While most of the significant effects were in the expected directions, some of these relationships were less intuitive. For example, participants’ SWB decreased as they got older (F(1.94, 37,989) = 25.39, p < 0.001), females reported significantly higher SWB than males (F(1,37,989) = 336.46, p < 0.001), and SWB increased with annual income up to 300,000 euros, and then decreases with incomes of greater than 350,000 euros (F(1.95, 37,989) = 13.37, p < 0.001).

### Rank-ordered categories

Because the number of predictors varied widely across categories, categorical contributions to predicting SWB were assessed using both the number and percentage of individual predictors within each category, as well as the average MSE ranking of these predictors (see Table 5). Of the 15 categories in this study, two (work environment and cognitive function) were not represented in the top 50 individual predictors, and four (future expectations, mental health, societal factors, and family status) had only one predictor on the list. Among the 13 categories represented, the number of predictors ranged from 1 to 7, the percentages ranged from 11–100%, and the average MSE ranking ranged from 9.17 to 40.67.

**Table 5:** Number, percentage and mean MSE rank of categories represented by the top 50 SWB predictors.

SWB predictor categories	Cat	N	N (%)	Mean rank
Social factors^a^	8	6	100	9.17
Future expectations	15	1	25	11.00
Physical health^a^	9	5	71	14.80
Demographics^a^	1	4	57	17.75
Financial status^a^	7	6	67	18.33
Personality^a^	14	5	100	22.00
Mental health	10	1	50	28.00
Health behaviors^a,b^	13	4	36	30.00
Living environment^b^	5	4	57	37.00
Childhood experiences^b^	4	7	78	37.86
Societal factors	3	1	20	38.00
Healthcare^b^	12	5	45	39.00
Family status	2	1	11	40.00

*Cat* category, *N* sample size, *N *(*%*) percentage of category predictors.

*Categories without a common superscript have significantly different mean MSE rankings (p < 0.05).

Social factors had the highest percentage (100%), highest mean rank (9.17), and the second-highest number of predictors (6), and all six social factors were among the top 17 individual predictors, including the top two. Five (71%) of the physical health predictors were among the top 50 SWB predictors—with three in the top 10—and the third-highest mean MSE ranking (16.80), while 5 (56%) of the nine measures of financial status were among the top 50—all of which were significant—with a mean rank of 18.33. Similarly, all five personality measures were among the top 50 predictors—with an average ranking of 22.00—although neuroticism had by far the strongest link to SWB, with over two times the MSE impact as the second strongest predictor (extraversion).

Health behaviors had 4 (36%) and healthcare had 6 (55%) predictors among the top 50, with average ranks of 30.00 and 36.83, respectively. And although childhood experiences had the largest number (7) and third-highest percentage of predictors (78%), it had only the tenth-highest mean ranking (37.86). Finally, living environment had the lowest average ranking (40.67), and among categories with multiple predictors on the list, the lowest number (3) and second-lowest percentage (43%) of the top 50 SWB predictors.

An omnibus Kruskal–Wallis test of categories with multiple rankings was found to be significant (H(12) = 27.21, p < 0.01), and individual Mann–Whitney comparisons revealed two tiers of categorial SWB predictors in terms of MSE ranking. The first tier included the seven highest-ranking categories, all of which were significantly higher than the five lowest-ranking categories (the 8th category, health behaviors, was not significantly different than either tier).

## Discussion

### SWB across countries

As expected, subjective well-being varied widely across countries in this research, and their rank order was very similar—though not identical—to that of the World Happiness Report (WHR) reported around the same time. This makes sense, given the conceptual overlap between happiness and subjective well-being, and it suggests that the SWB predictors in the current study may also help explain longstanding international differences in happiness, which could be tested more directly by adding WHR information to future SHARE surveys.

The current study also identified eight groups of nations that differed significantly in SWB, and found that Northern European countries reported significantly higher SWB than those in the South. These findings are consistent with previous findings^68^, and they indicate that any salutary effects of Southern Europe’s warmer, sunnier weather were eclipsed by other factors, which may include Northern Europe’s higher levels of basic services, economic prosperity, civic engagement and social cohesion^18,19,68^. These explanations can be examined more directly in future research comparing the predictive impact of each factor on SWB within each region. These findings also illustrate that while nominal rankings are interesting and important, significance testing can provide additional, empirically-meaningful information about differences between countries, regions, and other populations.

### Individual and categorical SWB predictors

In this representative sample of older European and Israeli adults, social factors were consistently the strongest determinants of subjective well-being at both the individual and group level. The top three social factors (loneliness, social activity satisfaction, social network satisfaction) were more qualitative social indices, while the bottom three were more quantitative measures (social contact frequency, social network distance, and social network size). These results are consistent with a large literature showing significant, positive relationships between social factors and SWB^69–71^, as well as a smaller but longstanding group of studies showing stronger effects of qualitative than quantitative social factors on SWB^62,72,73^.

As with social factors, the most qualitative health factor (general health) was also a stronger SWB predictor than the other, more quantitative health indices (e.g., number of chronic illnesses). This may be due to their shared qualitative nature, and/or the fact that, like SWB, qualitative measures also reflect broader, more general constructs. The latter hypothesis can be tested more directly in future research that simultaneously examines the impact of both general and more specific health evaluations (e.g., strength, mobility, cardiovascular fitness) on subjective well-being. Although body mass index (BMI) was negatively associated with SWB, this relationship was only marginally significant, suggesting the possibility that the adverse impact of BMI on subjective well-being may be more pronounced at higher BMIs. This could be assessed by comparing quartile or tertile splits, and/or by analyzing slope SWB across body mass index levels.

In this research, SWB decreased significantly as participants got older. This is in contrast to previous studies that have found a U-shaped curve relating age to SWB, whereby SWB decreases during young adulthood, bottoms out in middle age, and steadily increases later in life^69^. To the extent that SWB decreases with age, the current results suggest that it may largely be due to adverse changes in factors such as loneliness, social satisfaction, financial stress, physical limitations, chronic illness and/or injury, and impending mortality. It may also reflect older adults’ diminished mental, physical and/or social status relative to other, younger individuals—including, perhaps, their younger selves.

Gender differences in previous SWB studies have been mixed, and a recent meta-analysis of 281 effects sizes and over 1 million participants found no differences in life-satisfaction ratings between males and females^73^. The higher SWB among females in this research suggests that these older women may be less lonely and more socially satisfied, which would be consistent with prior research findings that older women reported larger social networks and more satisfaction with them^74^. These women may also be more positive, frugal, behaviorally healthy, physically active and—given that they tend to live longer—less concerned about their mortality, all of which can be examined in future studies using SHARE data and other similar research.

Financial status also had a significant, independent influence on participants’ SWB. However, the strongest financial predictor was not income, but rather being able to make ends meet, suggesting that the benefit of material wealth to subjective well-being is based more on sufficiency than maximization. This conclusion is further supported by the nonlinear relationship between income and SWB in this research, which was positive up to 350,000 euros/year, and negative at higher income levels. While also a significant, independent predictor, employment did not affect SWB as much as income, which is consistent with the fact that employment is more distal (and complex) than the income it produces. A financially-based lack of healthcare had a greater impact on SWB than a similar lack of heat, which was more impactful than periods of hunger. These results may reflect a valuation hierarchy for these amenities, and/or their respective frequencies, which may have increased their statistical power by reducing the skewness of their distributions.

Of the Big Five personality measures, neuroticism was the strongest SWB predictor. This is consistent with previous research, both in terms of the link between neuroticism and subjective well-being^75^, and its impact on SWB relative to the other Big-Five traits^40^. The greater impact of neuroticism may be due to its being the only Big-Five trait with a negative valence, as negative psychological experiences have been found to have stronger effects than positive ones of similar intensity^73^. Neuroticism also reflects a tendency to perceive and experience (i.e., internalize) things in a negative way, while the other Big-Five personality traits (openness, conscientiousness, agreeableness, and extraversion) are more externally directed, which may also reduce their relative impact on subjective experiences. These hypotheses can be examined more directly in future research that includes both positive and negative dispositional measures that are either internally or externally directed.

Although most of the childhood experiences were among the top 50 SWB predictors, they were not highly ranked. Interestingly, all three factors reflecting participants’ status at age 10 (rooms in the house, books, and math skills) ranked higher than the three physical-abuse factors (father, mother, nonparent). While this may reflect the long-term importance of childhood financial security, literacy and quantitative reasoning, it may also be that childhood abuse is more complex and difficult to define—and thus harder to report accurately. In addition, the stress and potential stigma often associated with being abused as a child may leave participants less able or willing to recount these experiences.

Fewer than half of the health behaviors were among the top 50 SWB predictors, and only two of these—moderate and vigorous exercise—were statistically significant. Given previous research showing, for example, a significant negative impact of cigarette use on QoL and SWB^53,54^, these results may reflect the multivariate nature of the current analyses, and its controlling for a large number of other factors (and their multicollinearity). It also suggests that at least some of these health behaviors may influence SWB indirectly through health outcomes. For example, alcohol consumption was significantly related to chronic illness, activity limitations, and perceived health, all of which were significantly correlated with SWB. These potential mechanisms can be further clarified by testing mediational models with these and similar other datasets. These health-behavior results also illustrate the difference between MSE and effect-size/significance testing, which, while highly correlated, are not identical metrics, and argues for the use of both when assessing the predictors of SWB and other outcomes.

In this study, healthcare factors were not strong predictors of subjective well-being. It may be that health-insurance status and the type of healthcare delivered (domestic vs. nursing care) are not as salient to people as the quality of the healthcare (or other factors), or that their effects may operate through other SWB predictors. Similarly, the relatively weak link between participants’ living environment and their subjective well-being suggests that while local connections, the help of others, and perceptions of crime are relevant, they are not as central to SWB as social, health, finances, demographic or personality factors.

Among the lone category representatives, expecting to be alive in 10 years was the strongest SWB predictor, which may reflect a greater salience of mortality to older than younger adults. Given its intuitive connection to subjective well-being, happiness might have been expected to rank higher as a predictor of SWB (#28). However, this may have been due to its being measured in terms of whether or not participants had experienced periods of happiness, rather than a continuous measure of their current happiness level. Rather than marital status, number of children, or parental relationships, whether participants looked after their grandchildren was the only family factor significantly related to SWB. This may reflect the lower levels of conflict and responsibility that many older adults experience with their grandchildren relative to their children. Frequency of prayer was the only societal factor among the top 50 SWB predictors, and while it approached statistical significance, it did not reach it. Moreover, the direction of prayer’s effect was negative, suggesting that rather than enhancing SWB, more prayer may be generated by lower subjective well-being and/or the factors associated with it.

### Strengths and limitations

This research included representative samples from 18 countries, totaling almost 38,000 participants and providing generalizability & sufficient statistical power to assess the independent effects of 94 individual factors and 15 categories on an aggregate measure of subjective well-being. In addition, combining machine learning and GAM enabled relatively unbiased, rank-ordered MSE scores, effect sizes & significance levels, as well as a direct comparison between these predictive indices.

To balance content validity and participant burden, a compound measure of SWB (combining life satisfaction, QoL and depression) was used in this and certain other SHARE research^58,59^. While this approach has important advantages, it also has a number of limitations. First, by combining different SWB elements, this research is unable to determine the impact of study predictors on any of these individual SWB components, nor can one make direct comparisons between previous single-component studies and the current findings. And although the current results are generally consistent with prior research using other SWB measures^37,59,63,76–82^, future research that includes both individual and aggregate indices would be able to address this issue more definitively.

The current SHARE SWB measure also does not contain a positive measure of experiential well-being (i.e., positive affect), restricting its representation of subjective well-being, and further limiting direct comparisons with other SWB research. This could be addressed by simply adding one or more measures of positive affect, and examining their individual and combined relationships with potential predictors. In addition, the self-reported, often retrospective nature of this research subjects these data to potential bias and other sources of error, which may help explain some of the null results. Although this limitation is inevitable for certain assessments among older adults (e.g., childhood experiences), objective measures and prospective analyses can address this issue in future longitudinal research.

While the current findings suggest certain mechanisms for the observed effects, the correlational analyses preclude any causal conclusions, although these mechanisms could be clarified by future research testing moderators and/or mediational models. The categorical analyses should also be interpreted with caution, for category rankings often depended on which (and how many) of the overlapping predictors were included in each. For example, including loneliness in mental health would make it the third highest-ranking category, and including hunger periods in physical health rather than financial factors would reverse their respective rankings. However, this further illustrates the importance of examining predictors at the individual level. Finally, because this research was conducted with older European and Israeli adults, the results may not generalize to other age groups or nationalities.

### Future research

Future multivariate modeling using SHARE and other data would be useful to test specific moderators and/or mediational mechanisms that may help explain the interrelationships between the current SWB predictors, and how they combine to determine subjective well-being. It may also be useful to employ more continuous independent measures, for over one-fourth of the prospective SWB predictors in this research were dichotomous, which may have reduced their predictive capacity. Moreover, given that many of the measures in this and similar surveys have significant positive or negative social connotations (e.g., health, illness, finances, social outcomes, SWB), it would be useful for future research to include social desirability to control for this potential confound.

## Conclusion

By addressing the multifaceted nature of subjective well-being and the analytic requirements for assessing its potential determinants, this research can further our understanding of what SWB is, how it is influenced, and what steps are most likely to maximize its development—especially among older adults. In so doing, it is hoped that this and similar research in different contexts can improve people’s subjective well-being, as well as its many related individual and societal benefits.

## Supplementary Information

Supplementary Information.

## Data Availability

The data supporting this finding are available at: https://gitlab.huma-num.fr/gveracruz/europeans_subjective_well_being.

## Abbreviations

CASP-19: 19-Item Quality of Life Scale
CASP-12: 12-Item Quality of Life Scale
EU: European Union
R^2^: Explained variance
GAM: Generalized additive modelling
%IncMSE: Percent increase in mean squared error
MSE: Mean squared error
QoL: Quality-of-life
REML: Restricted maximum likelihood
SHARE: Survey of Health, Ageing and Retirement in Europe
SWB: Subjective well-being
SWLS: Satisfaction with Life Scale
WHR: World Happiness Report
