# Supplementary material for: Using artificial intelligence to identify the top 50 independent predictors of subjective well-being in a multinational sample of 37,991 older European & Israeli adults
Source: Sci Rep. 2023 Jul 13;13:11352. doi: 10.1038/s41598-023-38337-w (PMC10344944; doi:10.1038/s41598-023-38337-w)
Supplement: Supplementary file 1 — Supplementary Information. [file 41598_2023_38337_MOESM1_ESM.docx]

**APPENDIX**

**Appendix A**. List of all variables (predictors and outcome) in the present study.

| Predictor Categories  (Study) | Predictor  Names  (Study) | Predictor  Descriptions/Scales  (Study) | Predictor  Descriptions/Scales  (SHARE) | Variable Codes  (SHARE) |
| --- | --- | --- | --- | --- |
| 1. Demographics | Country | Current country | Current country | country |
|  | Birth country | Birth country | Birth country | dn004 dn005_ |
|  | Age | Years of age | Years of age | age_int |
|  | Birth year | Birth year | Birth year | yrbirth |
|  | Sex | 0 (male); 1 (female) | 1 (male); 2 (female) | gender |
|  | Education | 0 (no education) to 6 (max education) | 0 (no education) to 6 (max education) | isced1997_r |
|  | Dominant hand | 1 (right); 2(left); 3(ambidexterity) | 1 (right); 2(left); 3(ambidexterity) | gs004_ |
| 2. Family Status | Marital status | 1 (never married);  2 (divorced); 3 (widowed);  4-6 (married) | 1-3 (married); 4 (never married); 5 (divorced);  6 (widowed) | dn014_ |
|  | Number of children | Number of children parented | Number of children parented | ch001_ |
|  | Relationship with mother | 1 (poor) to 5 (excellent) | 1 (excellent) to 5 (poor) | cc722_1 |
|  | Relationship with father | 1 (poor) to 5 (excellent) | 1 (excellent) to 5 (poor) | cc722_2 |
|  | Looked after grandchildren | 0 (no); 1 (yes) | 1 (yes); 5 (no) | sp014_ |
| 3. Societal  Factors | Political conservatism | 0 (extreme left) to  10 (extreme right) | 0 (extreme left) to  10 (extreme right) | ex028_ |
|  | Frequency of prayer | 1 (never) to 6 (daily) | 1 (daily) to 6 (never) | ex029_ |
|  | Mother suffered discrimination | 0 (no); 1 (yes) | 1 (yes); 5 (no) | gl738 |
|  | Father suffered discrimination | 0 (no); 1 (yes) | 1 (yes); 5 (no) | gl740 |
|  | Legal guardian suffered discrimination | 0 (no); 1 (yes) | 1 (yes); 5 (no) | gl022_ |
| 4. Childhood  Experiences | Rooms at age 10 | Rooms in house at age 10 | Rooms in house at age 10 | cc002_ |
|  | Books at age 10 | Number of books owned at age 10 | Number of books owned at age 10 | cc008_ |
|  | Math skills at age 10 | 1 (worse than average) to 5(better than average) | 1(better than average) to 5(worse than average) | cc010_ |
|  | Physical abuse as child (mother) | 1 (never); 2 (rarely);  3 (sometimes); 4 (often) | 1 (often); 2 (sometimes);  3 (rarely); 4 (never) | cc725_1 |
|  | Physical abuse as child (father) | 1 (never); 2 (rarely);  3 (sometimes); 4 (often) | 1 (often); 2 (sometimes);  3 (rarely); 4 (never) | cc725_2 |
|  | Physical abuse as child (nonparent) | 1 (never); 2 (rarely);  3 (sometimes); 4 (often) | 1 (often); 2 (sometimes);  3 (rarely); 4 (never) | cc727_ |
|  | Lonely in childhood | 1 (never); 2 (rarely);  3 (sometimes); 4 (often) | 1 (often); 2 (sometimes);  3 (rarely); 4 (never) | cc729_ |
|  | Comfortable friend group | 1 (never); 2 (rarely);  3 (sometimes); 4 (often) | 1 (often); 2 (sometimes);  3 (rarely); 4 (never) | cc730_ |
|  | Religion important as child | 1 (not important) to  4 (very important) | 1 (important) to 4 (not important) | cc728_ |
| 5. Living  Environment | Urban living | 1 (rural area); 2 (small town); 3 (large town);  4 (suburb); 5 (big city) | 1 (big city); 2 (suburbs); 3 (large town); 4 (small town); 5 (rural area) | iv009_ |
|  | Live in clean area | 1 (disagree) to 4 (agree) | 1 (agree) to 4 (disagree) | mn024_ |
|  | Local crime problem | 1 (disagree) to 4 (agree) | 1 (agree) to 4 (disagree) | hh024_ |
|  | Local help available | 1 (disagree) to 4 (agree) | 1 (agree) to 4 (disagree) | hh023_ |
|  | Others helpful | 1 (hardly ever);  2 (sometimes); 3 (usually);  4 (all the time) | 1 (all the time); 2 (usually); 3 (sometimes); 4 (hardly ever) | hh025_ |
|  | Receive efficient help | 1 (hardly ever); 2 (sometimes); 3 (usually);  4 (all the time) | 1 (all the time); 2 (usually); 3 (sometimes); 4 (hardly ever) | ph051_ |
|  | Feel local connection | 0 (disagree); 1 (agree) | 1 (agree) to 4 (disagree) | hh022_ |
| 6. Work  Environment | Employment status | 1 (retired); 2 (unemployed); 3 (permanent disability);  4 (homemaker);  5 (employed) | 1 (retired); 2 (employed); 3(unemployed); 4 (permanently sick or disabled); 5 (homemaker) | ep005_ |
|  | Job sector* | 14 kinds of business, industry or services | 14 kinds of business, industry or services | ep018_ |
|  | Job permanence | 0 (not working);  1 (temporary);  2 (permanent) | 1 (short-terms); 2 (permanent); 0 (don't work) | ep011_ |
| 7. Financial  Status | Income | PPP adjusted income | PPP adjusted income | nomx2015 pppc2015 thinc |
|  | Family well-off | 1 (poor); 2 (average);  3 (well off) | 1 (well off financially); 2 (about average); 3(poor); 4-5(other eg did not live with family) | cc733_ |
|  | Making ends meet | 1 (difficult) to  4 (easily) | 1 (difficult) to  4 (easily) | co007_ |
|  | Can pay unexpected bills | 0 (no); 1 (yes) | 1 (yes); 5 (no) | co206_ |
|  | Financial hardship periods | 0 (no); 1 (yes) | 1 (yes); 5 (no) | gl011_ |
|  | No heat due to cost | 0 (no); 1 (yes) | 1 (yes); 5 (no) | co209_ |
|  | No meds duet to cost | 0 (no); 1 (yes) | 1 (yes); 5 (no) | hc760_ |
|  | No healthcare due to cost | 0 (no); 1 (yes) | 1 (yes); 5 (no) | hc114_ |
|  | Hunger periods | 0 (no); 1 (yes) | 1 (yes); 5 (no) | gl014_ |
| 8. Social  Relationships | Social network size | Number of people in social network | Number of people in social network | sn_size_w6 |
|  | Social network distance | 1-3 (< 1km); 4-5 (< 25km); 6 (< 100km); 7-8 (>100km) | 1-3 (< 1km); 4-5 (< 25km); 6 (< 100km); 7-8 (> 100km) | prx_mean |
|  | Social contact frequency | 1 (never); 2 (annually); 3 (≈ once a month);4 (≈ twice a month); 5 (≈ once a week); 6 (≈ twice a week); 7 (daily) | 1 (daily); 2 (≈ two time per week); 3 (≈ once a week); 4 (≈ two time per month); 5 (≈ once per monthly); 6 (annually); 7 (never) | contact_mean |
|  | Social activity satisfaction | 0 (completely dissatisfied) to 10 (completely satisfied) | 0 (completely dissatisfied) to 10 (completely satisfied) | ac037_ ac038_ |
|  | Social network satisfaction | 0 (completely dissatisfied) to 10 (completely satisfied) | 0 (completely dissatisfied) to 10 (completely satisfied) | sn_satisfaction |
|  | Loneliness | 0 (not at all) to  6 (very lonely) | R-UCLA Loneliness Scale: 3 (not lonely) to 9 (very lonely) | loneliness |
| 9. Physical  Health | Body mass index | weight (kg) / height (m^2^) | weight (kg) / height (m^2^) | bmi |
|  | Self-rated general health | 1 (poor); 2 (fair); 3(quite good); 4 (good);  5 (excellent) | 1 (excellent); 2 (good); 3(quite good); 4 (fair); 5 (poor) | self_perceived_health |
|  | Number of chronic diseases | Number of current chronic diseases | Number of current chronic diseases | chronicw6c |
|  | Have a chronic illness | 0 (no); 1 (yes) | 1 (yes); 5 (no) | ph004_ |
|  | Limited activity due to health | 1 (not limited);  2 (not severely limited);  3 (severely limited) | 1 (severely limited); 2 (limited, but not severely); 3 (not limited) | ph005_ |
|  | Trouble with pain | 0 (no); 1 (yes) | 1 (yes); 5 (no) | ph084_ |
|  | Take five or more meds per day | 0 (no); 1 (yes) | 1 (yes); 5 (no) | ph082_ |
| 10. Mental  Health | Happiness periods | 0 (no); 1 (yes) | 1 (yes); 5 (no) | gl002_ |
|  | Stress periods | 0 (no); 1 (yes) | 1 (yes); 5 (no) | gl005_ |
| 11. Cognitive  Function | Word memory | number of words remembered | number of words remembered | cf016tot |
|  | Numeracy | 0 (bad) to 5 (good) | 0 (bad) to 5 (good) | numeracy2 |
|  | Time/date orientation | 0 (bad) to 5 (good) | 0 (bad) to 5 (good) | orienti |
| 12. Healthcare | Public insurance | 0 (no); 1 (yes) | 1 (yes); 5 (no) | hc116d1 |
|  | Private health insurance | 0 (no); 1 (yes) | 1 (yes); 5 (no) | hc116d3 |
|  | Private mandatory insurance | 0 (no); 1 (yes) | 1 (yes); 5 (no) | hc116d2 |
|  | Comprehensive insurance | 0 (no); 1 (yes) | 1 (yes); 5 (no) | hc113_ |
|  | Insurance satisfaction | 0 (dissatisfied); 1 (satisfied) | 1-2 (satisfied); 3-4 (dissatisfied) | hc125_ |
|  | Long-term health insurance | 0 (no); 1 (yes) | 1 (yes); 5 (no) | hc116dno |
|  | Life insurance | 0 (no); 1 (yes) | 1 (yes); 5 (no) | as067_ |
|  | Wait too long for doctor | 0 (no); 1 (yes) | 1 (yes); 5 (no) | hc115_ |
|  | Informal care given | 0 (no informal care);  1 (informal domestic);  2 (informal nursing);  3 (informal domestic  & domestic) | 0 (no informal care); 1 (informal domestic); 2 (informal nursing); 3 (both informal nursing and domestic) | sp010_ sp008_ |
|  | Informal care received | 0 (no informal care);  1 (informal domestic);  2 (informal nursing);  3 (informal domestic  & domestic) | 0 (no informal care); 1 (informal domestic); 2 (informal nursing); 3 (both informal nursing and domestic) | sp002_ sp004_ |
|  | Formal care received | 0 (no informal care);  1 (informal domestic);  2 (informal nursing);  3 (informal domestic  & domestic) | 0 (no formal care); 1 (formal domestic); 2 (formal nursing); 3 (both formal nursing and domestic) | hc127 |
| 13. Health  Behaviors | Daily smoker | 0 (no); 1 (yes) | 1 (yes); 5 (no) | br001_ |
|  | Still smoking | 0 (no); 1 (yes) | 1 (yes); 5 (no) | br002_ |
|  | Cigarettes per day | Number of cigarettes smoked per day | Number of cigarettes smoked per day | br006_ |
|  | Alcohol in last 7 days | 0 (no); 1 (yes) | 1 (yes); 5 (no) | br039_ |
|  | Number of drinks in last 7 days | Number of alcoholic drinks in the last 7 days | Number of alcoholic drinks in the last 7 days | br040_ |
|  | Moderate physical activity | 1 (never/almost never);  2 (monthly); 3 (weekly);  4 (daily) | 1 (daily); 2 (weekly); 3 (monthly); 4 (hardly ever or never) | br016_ |
|  | Vigorous physical activity | 1 (never/almost never);  2 (monthly); 3 (weekly);  4 (daily) | 1 (daily); 2 (weekly); 3 (monthly); 4 (hardly ever or never) | br015_ |
|  | Fruit & vegetable consumption | 1 (less than once a week);  2 (once a week);  3 (two times a week);  4 (three times a week);  5 (daily) | 1 (every day); 2 (three times per week; 3 (two times per week); 4 (once a week); 5 (less) | br029_ |
|  | Meat consumption | 1 (less than once a week);  2 (once a week);  3 (two times a week);  4 (three times a week);  5 (daily) | 1 (every day); 2 (three times per week; 3 (two times per week); 4 (once a week); 5 (less) | br028_ |
|  | Dairy consumption | 1 (less than once a week);  2 (once a week);  3 (two times a week);  4 (three times a week);  5 (daily) | 1 (every day); 2 (three times per week; 3 (two times per week); 4 (once a week); 5 (less) | br026_ |
|  | Legume & egg consumption | 1 (less than once a week);  2 (once a week);  3 (two times a week);  4 (three times a week);  5 (daily) | 1 (every day); 2 (three times per week; 3 (two times per week); 4 (once a week); 5 (less) | br027_ |
| 14. Personality | Neuroticism | 1 (low) to 5 (high) | 1 (low) to 5 (high) | bfi10_neuro |
|  | Extraversion | 1 (low) to 5 (high) | 1 (low) to 5 (high) | bfi10_extra |
|  | Agreeableness | 1 (low) to 5 (high) | 1 (low) to 5 (high) | bfi10_agree |
|  | Openness to experience | 1 (low) to 5 (high) | 1 (low) to 5 (high) | bfi10_open |
|  | Conscientiousness | 1 (low) to 5 (high) | 1 (low) to 5 (high) | bfi10_consc |
| 15. Future  Expectations | Alive in 10 years | 0 to 100 | 0 to 100 | ex709_ |
|  | Pension reduction expectations | 0 to 100 | 0 to 100 | ex007_ |
|  | Pension increase expectations | 0 to 100 | 0 to 100 | ex008_ |
|  | Trust in others | 0 to 100 | 0 to 100 | ex026_ |
| Outcome Category  (Study) | Outcome  Name  (Study) | Outcome  Descriptions/Scales  (Study) | Outcomes  Descriptions/Scales  (SHARE) | Variable Codes  (SHARE) |
| Subjective  Well-being | Subjective  Well-being | Depression - 0 (not at all) to 12 (very depressed) | scale 0 (not depressed) to 12 (very depressed) | eurod |
|  |  | Quality of life - 12 (low) to 48 (high) | scale 12 (low life quality) to 48 (high life quality) | casp |
|  |  | Life satisfaction – 0 (low) to 10 (high) | continuous scale 0-10 point | ac012_ |

Note: While expressed in Euros, the income variable is indexed for both nominal exchange rates (ner) and purchasing power parity (ppp) to control for differences in both currency values and standards of living.

*1 (Agriculture, hunting, forestry, fishing); 2 (Mining and quarrying); 3 (Manufacturing); 4 (Electricity, gas and water supply); 5 (Construction); 6 (Wholesale and retail trade; repair of motor vehicles, motorcycles and personal and household goods); 7 (Hotels and restaurants); 8 (Transport, storage and communication); 9 (Financial intermediation); 10 (Real estate, renting and business activities); 11 (Public administration and defence; compulsory social security); 12 (Education); 13 (Health and social work); 14 (Other community, social and personal service activities).

**Appendix B**. Frequency distributions for categorical study variables.

| SWB Predictors | Rank | Cat | N | Values (Labels) | Frequency distribution per category |
| --- | --- | --- | --- | --- | --- |
| Loneliness | 1 | 8 | 37991 | 0 (not at all) to  6 (very lonely) | 0=59.1%  1=18.3%  2=9.8%  3=7.3%  4=2.8%  5=1.3%  6=1.5% |
| Social activity satisfaction | 2 | 8 | 37991 | 0 (completely dissatisfied) to 10 (completely satisfied) | 0=1.3%  1=.3%  2=.6%  3=.8%  4=1.0%  5=7.2%  6=4.8%  7=10.9%  8=32.1%  9=15.9%  10=25.2% |
| Self-rated general health | 3 | 9 | 37991 | 1 (poor); 2 (fair); 3(quite good); 4 (good); 5 (excellent) | 1=10.2%  2=28.5%  3=36%  4=18.0%  5=7.3% |
| Making ends meet | 4 | 7 | 37991 | 1 (difficult) to  4 (easily) | 1=8%  2=17.8%  3=51.2%  4=23% |
| Country | 5 | 1 | 37991 | n/a | n/a |
| Social network satisfaction | 7 | 8 | 37991 | 0 (completely dissatisfied) to 10 (completely satisfied) | 0=0.7%  1=1.1%  2=1.1%  3=3.2%  4=2.1%  5=3.5%  6=3.4%  7=6.8%  8=18.8%  9=19.6%  10=40.5% |
| Limited activity due to health | 9 | 9 | 37991 | 1 (not limited);  2 (not severely limited);  3 (severely limited) | 1=54.0%  2=30.9%  3=15.1% |
| No healthcare due to cost | 14 | 7 | 37991 | Y/N | 13.8%Y |
| Social network size | 17 | 8 | 37991 | Number of people in social network | 0=4.1%  1=24.8%  2=23.7%  3=20.8%  4=13.2%  5=7.8%  6=4.0%  7=2.5% |
| Employment status | 18 | 7 | 37991 | 1 (retired); 2 (unemployed); 3 (permanent disability);  4 (homemaker);  5 (employed) | 1=58.6%  2=3.0%  3=3.1%  4=7.5%  5=27.9% |
| Vigorous physical activity | 19 | 13 | 37991 | 1 (never/almost never);  2 (monthly); 3 (weekly);  4 (daily) | 1=40.9%  2=9.2%  3=14%  4=35.9% |
| No heat due to cost | 20 | 7 | 37991 | Y/N | 6.9%Y |
| Have a chronic illness | 21 | 9 | 37991 | Y/N | 52.9%Y |
| Sex | 22 | 1 | 37991 | F/M | ­­56.4%F |
| Moderate physical activity | 25 | 13 | 37991 | 1 (never/almost never);  2 (monthly); 3 (weekly);  4 (daily) | 1=11.1%  2=5.2%  3=12.8%  4=70.9% |
| Others helpful | 26 | 12 | 37991 | 1 (hardly ever);  2 (sometimes); 3 (usually);  4 (all the time) | 1=1.8%  2=6.2%  3=65.1%  4=26.9% |
| Happiness periods | 28 | 10 | 37991 | Y/N | 44.4%Y |
| Education | 29 | 1 | 37991 | 0 (no education) to 6 (max education) | 0=4.3%  1=14.3%  2=18.2%  3=35%  4=5%  5=22.3%  6=0.9% |
| Comprehensive insurance | 31 | 12 | 37991 | Y/N | 40.2%Y |
| Feel local connection | 32 | 5 | 37991 | 1 (disagree) to 4 (agree) | 1=1%  2=3.5%  3=25.3%  4=70.3% |
| Long-term health insurance | 35 | 12 | 37991 | Y/N | 68.0%Y |
| Lonely in childhood | 36 | 4 | 37991 | 1 (never); 2 (rarely);  3 (sometimes); 4 (often) | 1=59.8%  2=19.6%  3=14%  4=6.6% |
| Informal care given | 37 | 12 | 37991 | 0 (no informal care);  1 (informal domestic);  2 (informal nursing);  3 (informal domestic  & nursing) | 0=4.4%  1=2.1%  2=20%  3=73.5% |
| Frequency of prayer | 38 | 3 | 37991 | 1 (never) to 6 (daily) | 1=43.8%  2=17.2%  3=7.4%  4=7.3%  5=15.8%  6=8.5% |
| Math skills at age 10 | 39 | 4 | 37991 | 1 (worse than average) to 5(better than average) | 1=4%  2=10.8%  3=53.5%  4=22.4%  5=10.2% |
| Looked after grandchildren | 40 | 2 | 37991 | Y/N | 21.5%Y |
| Local help available | 42 | 5 | 37991 | 1 (disagree) to 4 (agree) | 1=1.8%  2=6.2%  3=65.1%  4=26.9% |
| Physical abuse as child (father) | 43 | 4 | 37991 | 1 (never); 2 (rarely);  3 (sometimes); 4 (often) | 1=64.6%  2=21.7%  3=10.6%  4=3.1% |
| Physical abuse as child (nonparent) | 44 | 4 | 37991 | 1 (never); 2 (rarely);  3 (sometimes); 4 (often) | 1=82.7%  2=10.8%  3=5%  4=1.6% |
| Informal care received | 45 | 12 | 37991 | 0 (no informal care);  1 (informal domestic);  2 (informal nursing);  3 (informal domestic  & nursing) | 0=2.5%  1=.7%  2=17%  3=79.8% |
| Hunger periods | 46 | 7 | 37991 | Y/N | 5.3%Y |
| Private health insurance | 47 | 12 | 37991 | Y/N | 7.4%Y |
| Local crime a problem | 48 | 5 | 37991 | 1 (disagree) to 4 (agree) | 1=28.4%  2=59%  3=9.5%  4=3.1% |
| Dairy consumption | 49 | 13 | 37991 | 1 (less than once a week);  2 (once a week);  3 (two times per week); 4 (three times per week); 5 (daily) | 1=4.1%  2=3.6%  3=7.6%  4=18.3%  5=66.4% |
| Physical abuse as child (mother) | 50 | 4 | 37991 | 1 (never); 2 (rarely);  3 (sometimes); 4 (often) | 1=61.8%  2=21.3%  3=13.1%  4=3.8% |

**Appendix C.** Reference list related to citations in Table 1

| Benjamin DJ, Cooper KB, Feffetz O, Kimball M. (2017) Challenges in constructing a survey-based well-being index. Am Econ Rev.2017;107:81–85.  CDC Centers for Disease Control and Prevention (CDC; October 31). Well-being concepts;  2018. Available at: https://www.cdc.gov/hrqol/wellbeing.htm#three |
| --- |
|  |
| [Das KV, Jones-Harrell C, Fan Y, Ramaswami A, Orlove B, Botchwey N. Understanding subjective well-being: perspectives from psychology and public health. Public Health Rev.2020;41: 25. DOI: 10.1186/s40985-020-00142-4](https://doi.org/10.1186/s40985-020-00142-4) |
| Diener E, Oishi S, Tay L. Advances in subjective well-being research. Nat Hum Behav.2018;2(4): 253-260. DOI: 10.1038/s41562-018-0307-6 |
| Diener E, Wirtz D, Tov W, at al. (2009). New measures of well-being: Flourishing and positive and negative feelings. Soc Indic Res.2009;39(2): 247–266. DOI:10.1007/s11205-008-9169-1 |
| Diener E. Subjective well-being. Psychol Bull.1984;95(3):542-575. DOI: 10.1037/0033-2909.95.3.542 |
| Dolan P, Metcalfe R. Measuring subjective well-being: recommendations on measures for use by national governments. J Soc Policy.2012;41:409–427. DOI: 10.1017/S0047279411000833 |
| Dolan P, Peasgood T, White M. Measuring well-being for public policy: Lessons from economics. Oxf Rev Econ Policy.2008;24(2):249-276. |
| Easterlin RA. Will raising the incomes of all increase the happiness of all. J Econ Behav Organ.1995;27(1):35-47. DOI: 10.1016/0167-2681(95)00003-B |
| Frey BS, Stutzer A. Happiness and economics. Princeton, N.J.: Princeton University Press; 2002. |
| George LK. Still happy after all these years: Research frontiers on subjective well-being in later life. J Gerontol B Psychol Sci Soc Sci.2010;65(3): 331-339. |
| Gillham J, Adams-Deutsch Z, Werner J, et al. Character strengths predict subjective well-being during adolescence. J Posit Psychol.2011;6(1):31-44. |
| Goodman, F. R., Disabato, D. J., Kashdan, T. B., Kauffman, S. B. (2018). Measuring well-being: A comparison of subjective well-being and PERMA. The Journal of Positive Psychology, 13(4), 321-332.  Jovanović V, Lazić M. Is longer always better? A comparison of the validity of single-item versus multiple-item measures of life satisfaction. Appl Res Qual Life.2020;15(3), 675-692. DOI: 10.1007/s11482-018-9680-6 |
| Kahneman D, Krueger AB. Developments in the measurement of subjective well-being. J Econ Perspect.2006;20(1):3-24. DOI: 10.1257/089533006776526030 |
| Kashdan TB. The assessment of subjective well-being (issues raised by the Oxford Happiness Questionnaire). Pers Individ Differ.2004;36(5):1225–1232. DOI: 10.1016/S0191-8869(03)00213-7 |
| Kelley J, Evans MD. Societal Inequality and individual subjective well-being: Results from 68 societies and over 200,000 individuals, 1981–2008. Soc Sci Res.2017;62: 1-23. DOI: 10.1016/j.ssresearch.2016.04.020 |
| Lyubomirsky SONJA, Dickerhoof RENE. Subjective well-being. Handbook of girls’ and women’s psychological health: Gender and wellbeing across the life span; 2005: 166-174. |
| Odermatt R; Stutzer A. Subjective well-being and public policy. In: Diener, E. et al. (eds.) Handbook of wellbeing: 954–968. DEF Publishers; 2018. |
| Pérez-Rojo G, Martín N, Noriega C, López J. Psychometric properties of the CASP-12 in a Spanish older community dwelling sample. Aging Ment Health. 2018;22(5):5700-708. DOI: 10.1080/13607863.2017.1292208 |
| Prince MJ, Reischies F, Beekman AT, et al. Development of the EURO-D scale – a European, Union initiative to compare symptoms of depression in 14 European centres. Br J Psychiatry. 1999;174: 330-338. DOI: 10.1192/bjp.174.4.330 |
| Ryff CD, Keyes CLM. The structure of psychological well-being revisited. J Pers Soc Psychol.1995;69(4):719–727. DOI: 10.1037/0022-3514.69.4.719 |
| Veenhoven R. Sociological theories of subjective well-being. In: Eid M, Larsen RJ (eds). The science of subjective well-being. New York: Guilford Press; 2008:44–61 |
| Vik MH, Carlquist E. Measuring subjective well-being for policy purposes: The example of well-being indicators in the WHO “Health 2020” framework. Scand J Public Health.2018;46(2):279-286. DOI: 10.1177/1403494817724952 |
|  |
|  |
|  |
|  |
|  |
|  |
|  |
|  |
|  |
|  |
|  |
|  |
|  |
|  |
